# Biomedical data repositories require governance for artificial intelligence/machine learning applications at every step

**DOI:** 10.1093/jamiaopen/ooaf134

**Published:** 2025-12-01

**Authors:** Ellen Wright Clayton, Susannah Rose, Camille Nebeker, Laurie Novak, Yael Bensoussan, You Chen, Benjamin X Collins, Ashley Cordes, Barbara J Evans, Kadija S Ferryman, Samantha Hurst, Xiaoqian Jiang, Aaron Y Lee, Shannon McWeeney, Jillian Parker, Jean-Christophe Bélisle-Pipon, Eric Rosenthal, Zhijun Yin, Joseph Yracheta, Bradley Adam Malin, Bridge2AI Ethics Working Group, Nicholas Greig Evans, Subhashini Chandrasekharan, Ilana Goldberg, Barbara J Evans, Samantha Hurst, Shannon K McWeeney, Aaron Y Lee

**Affiliations:** Center for Biomedical Ethics and Society, Vanderbilt University Medical Center, Nashville, TN 37203, United States; Department of Biomedical Informatics, Vanderbilt University Medical Center, Nashville, TN 37203, United States; Herbert Wertheim School of Public Health & Human Longevity Science, University of California San Diego, San Diego, CA 92092, United States; Department of Biomedical Informatics, Vanderbilt University Medical Center, Nashville, TN 37203, United States; Department of Otolaryngology, Head & Neck Surgery, University of South Florida Morsani College of Medicine, Tampa, FL 33612, United States; Department of Biomedical Informatics, Vanderbilt University Medical Center, Nashville, TN 37203, United States; Department of Biomedical Informatics, Vanderbilt University Medical Center, Nashville, TN 37203, United States; Indigenous Media Studies in ENVS and Data Science, University of Oregon, Eugene, OR 97403, United States; School of Law, University of Florida, Gainesville, FL 32611, United States; Department of Health Policy and Management, Johns Hopkins Bloomberg School of Public Health, Baltimore, MD 21205, United States; Herbert Wertheim School of Public Health & Human Longevity Science, University of California San Diego, San Diego, CA 92092, United States; Department of Health Data Science and Artificial Intelligence, McWilliams School, University of Texas Health Science Center at Houston, Houston, TX 77030, United States; Department of Ophthalmology, University of Washington, Seattle, WA 98195, United States; Cancer Data Science, Oregon Health Science University Knight Cancer Institute, Portland, OR 97239, United States; Department of Medicine, University of California San Diego, San Diego, CA 92092, United States; Faculty of Health Sciences, Simon Fraser University, Burnaby, BC V5A 1S6, Canada; Division of Neurocritical Care, Harvard Medical School, Boston, MA 02114, United States; Department of Biomedical Informatics, Vanderbilt University Medical Center, Nashville, TN 37203, United States; Native BioData Consortium, Eagle Butte, SD 57625, United States; Department of Biomedical Informatics, Vanderbilt University Medical Center, Nashville, TN 37203, United States

**Keywords:** governance, data privacy, informed consent, data access

## Abstract

**Objectives:**

The NIH’s Bridge2AI Program has funded 4 “new flagship biomedical and behavioral datasets that are properly documented and ready for use with AI [artificial intelligence] or ML [machine learning] technologies” to promote the adoption of AI. This article discusses the challenges and lessons learned in data collection and governance to ensure their responsible use.

**Materials and Methods:**

We outline major steps involved in creating and using these datasets in ethically acceptable ways, including (1) data selection—what data are being selected and why, (2) increasing attention to public concerns, (3) the role of participant consent depending on data source, (4) ensuring responsible use, (5) where and how data are stored, (6) what control participants have over data sharing, (7) data access, and (8) data download.

**Results:**

We discuss ethical, legal, social, and practical challenges raised at each step of creating AI-ready datasets, noting the importance of addressing issues of future data storage and use. We identify some of the many choices that these projects have made, including how to incorporate public input, where to store data, and defining criteria for access to and downloading data.

**Discussion:**

The processes involved in the establishment and governance of the Bridge2AI datasets vary widely but have common elements, suggesting opportunities for future programs to lean upon Bridge2AI strategies.

**Conclusions:**

This article discusses the challenges and lessons learned in data collection and governance to ensure their responsible use, particularly as confronted by the 4 distinct projects funded by this program.

The NIH’s Bridge2AI Program seeks to generate “new flagship biomedical and behavioral datasets that are properly documented and ready for use with AI or ML technologies” to promote the adoption of AI for biomedical research and healthcare in the United States.^1^ It, therefore, funded creation of 4 very different biomedical datasets, collectively referred to as Data Generation Projects (DGPs), that are ready to be used to develop artificial intelligence (AI) and machine learning (ML): (1) Precision Public Health (Voice) collects voice data linked to health conditions, (2) Cell Maps for AI (CM4AI) focuses on spatiotemporal data derived from canonical cell lines, (3) Program in Clinical Care AI through the CHoRUS Network gathers multimodal data to improve recovery from acute and critical illness, and (4) Artificial Intelligence Ready and Equitable Atlas for Diabetes Insights (AI-READI) program is developing a multimodal dataset, particularly focusing on retinal imaging, for type 2 diabetes mellitus research to identify contributors to health outcomes.^2^ To maximize their utility for other investigators, these datasets are being created without specific hypotheses in mind for future AI model development. The Program must ensure that these datasets are FAIR (findable, accessible, interoperable, and reusable) and promulgate best practices for the development and use of these data. While some issues in collection and governance of data overlap across Bridge2AI, the diversity of these DGPs means that each presents its own unique challenges.

In the United States, the primary decision-makers about the collection and use of data for AI in healthcare include, but are not limited to, healthcare institutions, scientific investigators, regulators, sponsors, payers, and commercial entities. The roles and authority of each of these actors are shaped by law, patterns of practice, and various incentives, yet significant gaps in data governance to ensure responsible collection and use remain. In particular, patients and patient advocacy groups are increasingly significant stakeholders, especially regarding consent, data sharing, and use. In this paper, we outline major steps in creating the data used for the AI development process, using Bridge2AI DGPs for illustration, with a specific focus on data governance, or how data are and should be collected and used, addressing the challenges of each step. These include identifying who the decision makers usually are and who they ought to be, what should influence their choices, including US laws and policies, ethical principles that expand on respect for persons, beneficence, and justice^3^ to focus on transparency, promoting trust, and ensuring that all patients can benefit from these tools.^4–6^ These decisions must consider how data collected for one purpose may be used for another, especially in light of data sharing requirements. When appropriate, we suggest paths forward. This process highlights the critical need for transparency, feedback, safety, and robustness of data stewardship and provenance to promote trustworthiness of clinical and biomedical research AI.

## A brief description of the steps of data sourcing and governance

**Figure 1.**
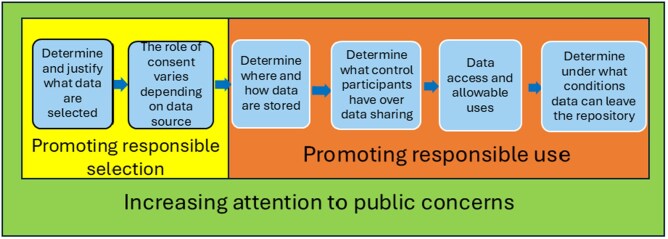
Steps in governance of data collection and decision-making and responsible use for the development of AI with greater attention to public concerns throughout. The first 2 steps—promoting responsible selection—address the primary work of the DGPs, while the remaining 4 steps—promoting responsible use—are crucial factors the DGPs must consider.

In this section, we describe 6 core steps in the data collection and data use and re-use for AI model development, as shown in Figure 1, addressing the governance issues of each. While the DGPs in Bridge2AI focus on data collection, their work must be informed by the need to promote responsible use and to attend to public concerns.

### Promoting responsible collection

Collecting data has ethical implications, which influence and should be influenced by every subsequent step and which must be addressed at the inception of a project, a process often conceptualized as “ethics by design.”^7^ This process begins by deciding what data to collect and why and what people need to know and what decisions they can and need to make about the use of data about them.

### Determine and justify what data are selected

In choosing which projects to fund, research and development sponsors—typically federal agencies and foundations and their respective program officers—assess which data should be sourced and set the conditions, which are specified in the request for applications, regarding how these projects can proceed. This plan is then interpreted by researchers and their home institutions.

The choices about data selection can be shaped by numerous factors, including the availability of resources, data quality as assessed by technical standards (FHIR, OMOP CDM, and GA4GH) that provide frameworks and tools that facilitate responsible and efficient data management, and comparability, as well as in most cases, the research hypotheses and study design.^4^ All these issues should be addressed and documented before data are amassed since it is difficult to respond to concerns that arise once projects have begun.^8^ Bridge2AI poses particular challenges in this regard in that data are not collected to investigate specific hypotheses, making it difficult to pre-specify which data will be needed for later users of the 3 clinically based Bridge2AI datasets. Thus, AI-READI casts a wide net, collecting responses to several surveys, a variety of clinical and imaging data, glucose and physical activity monitoring, and environmental measurements.^2^

### Increasing attention to public concerns

Seeking ongoing input from investigators, clinicians, and patients at all steps of research, from data generation to use, is increasingly encouraged, represented here as a cross-cutting issue.^9^^,^^10^ Yet, patients, research participants,^11^ and, to a lesser extent, clinicians have generally had little voice in the governance of research data despite increasingly urgent calls to engage with people who may be affected by data collection and use.^12^ Debates continue about how much control individuals should have over data about themselves. Thus, the Bridge2AI program incorporated an ethics pillar to increase community input, informed by empirical studies by the DGPs on a variety of issues such as data sharing, privacy, trust, and ethics. This work has included questionnaires,^13^ stakeholder forums at meetings,^13^ focus groups across diverse demographics, including those who have historically limited access to health care, including indigenous groups as well as Tribal colleges and historically black colleges and universities (HBCUs), panels,^14^ broader surveys, Delphi processes, datathons, and citizen science programs. For example, concerns expressed in a recent BRIDGE2AI DGP stakeholder forum included accuracy, fairness, nondiscrimination, and protection of privacy.^15^

### The role of participant consent varies depending on data source

Data for research can be collected from various sources and in numerous ways. These can broadly be grouped into (1) studies with direct patient interaction, which typically require prospective informed consent, and (2) studies without direct patient contact, which are typically retrospective and legally often do not require consent but do require strict compliance with federal and state laws defining specific conditions under which consent can be forgone.^16^

When data are collected directly from individuals, potential participants are informed about what data will be collected, which can include both retrospective (eg, from existing electronic health records) and new information (eg, from wearable sensors, surveys, research tests), stored, and used before they provide informed consent. This is the strategy undertaken by AI-READI and Voice. These projects are subject to the requirements of the Regulations for the Protection of Human Research Participants (ie, the Common Rule)^17^ as interpreted by local Institutional Review Boards (IRBs), which often vary in interpreting the regulations, in particular regarding whether consent and IRB oversight is required.^18^

By contrast, studies not requiring direct patient interaction, typically those that are conducted using data collected for other purposes—so-called secondary use—are sometimes exempt from consent and IRB oversight, particularly if the data are de-identified. For example, the Common Rule^17^ exempts large amounts of clinical data from its coverage and oversight entirely (eg, 45 CFR §§ 46.102(e) & 104(4)). The Health Insurance Portability and Accountability Act (HIPAA) and its Privacy Rule (45 C.F.R. parts 160, 164)^19^^,^^20^ applies only to identifiable data collected by “covered entities” and their “business associates” as these terms are defined by HIPAA and does not apply to those data that meet its de-identification standard. CHoRUS, which involves the collection and harmonization of existing clinical data, which are de-identified to the extent practicable and are stored in a controlled access repository where data cannot be exported (downloaded) and will be subject to use restrictions, has been deemed to pose “minimal risk” and so do not require consent.^16^ Additionally, the cell lines used for CM4AI were collected decades ago and are de-identified and so do not require consent or IRB oversight.

In other settings, some have argued that consent for research should still be sought from the individuals to whom the data pertain.^21^ Others, however, worry that consent itself offers little real privacy protection to participants and thus is never sufficient justification on its own for broad data sharing.^22^ Moreover, requiring specific consent in all cases can undermine the generalizability of datasets to all populations if participant uptake rates differ across demographics as they often do.^23–26^ Lack of uniformity of data obtained if consent is required may compound the fact that preexisting clinical data also frequently fail to reflect the health of the entire population due to disparate actions by health professionals, organizational processes, billing requirements, health care funding, and sociocultural factors.^27^ These gaps in representativeness need to be identified and mitigated to the extent possible in all research in support of AI in healthcare.

### *Promoting responsible use* ^28^

All involved in data collection and utilization and their institutions have ethical obligations to promote responsible use of data, another cross-cutting issue that involves several discrete steps. Thus, researchers must assess the nature of the data collected and consider what steps are needed based on the funding requirements and the scientific needs, including metadata standards, data quality, and harmonization across repositories, to ensure valid research results, balancing those factors with risks to research participants, including concerns about privacy or stigmatization.

### Determine where and how data are stored

People generally have more trust in how well their clinicians and the institutions where they receive care protect data about them than they do in the government, employers, insurers, and commercial entities, such as pharmaceutical companies.^29^ Thus, a data repository can boost its credibility by highlighting its link to the sites where participants seek clinical care.^30^

At the same time, where data go and how they are stored has evolved rapidly over the years. The simplest approach is to store data where they were initially collected in a locally controlled server, or what is referred to as on-premises storage. This approach dramatically reduces the amount of money spent on building and analyzing AI models, but it requires paying for acquiring computational infrastructure and staff to support it. Moreover, data holders may not always be up to date on the latest security threats and processes to mitigate them.^31^

Alternatively, as cloud computing has become easier to access and use, organizations have migrated their data into commercially managed environments (eg, Google Cloud, Microsoft Azure, and Amazon AWS). These strategies have various benefits and drawbacks, and the best fit for a dataset depends on a complex interplay of budget, risk tolerance, and computational flexibility. While cloud computing environments are typically maintained by well-trained security professionals, providing support for hundreds of thousands of users and data repositories, this protection comes at the cost of variable costs in computing, such that the typical process of iteratively refining an AI model can become extremely expensive,^32^ especially when deep learning is used.

It is also important to consider how such data are analyzed. Data in on-premises or cloud settings often can be analyzed only in their own environment, which can limit analytic power and generalizability of findings. In recent years, analytic strategies have emerged to overcome such siloed investigations. For example, federated learning allows the creation of AI models without moving data from its original site. Instead, only aggregated statistics or model parameters are shared. This approach can be reassuring to participants from a data privacy perspective. However, federated learning presents its own challenges—particularly, its performance may degrade when data across sites are not independent and identically distributed (non-IID). In such cases, model accuracy and convergence can suffer because local models are trained on disparate data distributions. Nevertheless, researchers have developed various techniques to mitigate these issues, such as advanced aggregation methods, sharing small portions of data, and modification of training algorithms to enhance robustness and accuracy despite data heterogeneity.^33^ Within Bridge2AI, AI-READI collaborates with Argonne National Laboratory on the use of PALISADE-X (Privacy-Preserving Analysis and Learning in Secure and Distributed Enclaves and Exascale Systems), the privacy preserving federated learning framework developed by the Department of Energy. Restricted access research environments that limit the risk of re-identification and privacy-preserving tools such as differential privacy are other approaches that have been advocated,^34^ but each has its own benefits and challenges.^32^

The DGPs vary in where they store the data they have collected and generated for dissemination. The data for CM4AI are completely open to the public on the University of Virginia Dataverse website (https://dataverse.lib.virginia.edu/dataset.xhtml?persistentId=doi:10.18130/V3/DXWOS5). The data from AI-READI require a data license for access and are available at FAIRhub at https://fairhub.io/datasets/2.^2^ All users must register and be authenticated. For diabetes research, users can download the datasets from the download portal after it is watermarked. For non-diabetes research and for controlled-access variables, the requests are reviewed by a Data Access Committee, and a data use agreement is required. CHoRUS devised a 3-enclave approach (analytics, tooling and governance) that addresses the anticipated diversity of researcher profiles and analytic needs and that ingests and combines large volumes of data from a heterogeneous network of 14 healthcare centers (for more detail, see: https://chorus4ai.org/documentation/).

### Determine what control participants have over data sharing

The NIH has long encouraged,^35^ and often required,^36^ broad data sharing by its grantees, subject only to exceptions granted on a case-by-case basis.^37^ A funding opportunity may require the data collected to be accessible to the public or only to investigators who meet certain qualifications and agree to certain limits on use. Research participants usually must agree to the mandated data sharing plan. Collections of pre-existing data that are exempt from consent and IRB oversight are also subject to funder-directed sharing. The datasets funded by Bridge2AI are required to be broadly accessible since they are being collected for use by other investigators.

While many people endorse data sharing to promote health research and discovery,^38^ this requirement raises concerns from some potential participants,^39^ particularly those who may be vulnerable to stigmatization from research or unintentional privacy breaches.^40^ Although laws providing Certificates of Confidentiality (CoCs) to protect research data now apply to all federally funded research,^41^ some participants may worry that they and people for whom they care may be harmed by the use of research data about them,^39^ particularly regarding psychiatric disorders and drug use as well as reproductive and transgender care.^42^ Their concerns have many causes, including a lack of knowledge about or trust in CoCs and confusion about conflicting federal^17^^,^^37^^,^^43^ and state data privacy laws. Even so, some people who are potentially at risk of privacy breaches or other harms nonetheless favor research participation because they believe that the potential benefits outweigh privacy threats.^44^^,^^45^

### Data access and allowable uses

Decisions about data access have at least 3 parts. These include defining (1) who makes decisions about access, (2) who can access the data for which purposes, and (3) how much access users have. Answering these questions requires weighing the benefits of a specific data use and the potential risks to the individuals to whom the data pertain, which may increase as data sharing increases in scale and complexity.

Data Access Committees typically decide who can use data, but their authority and their actions are shaped by where repositories are located, their governance structures, and their ability to provide safe, secure, and accountable management. Repositories located within federal agencies, such as the NIH’s Database of Genotypes and Phenotypes (dbGaP), by law, for example, are governed only by federal employees.^46^ Thus, decisions about where to locate repositories are changing to enable public involvement. In addition, some assert that individuals reflected in a dataset should be able to control who has access to the data about them on an ongoing basis,^21^ which can be challenging. Furthermore, indigenous groups as sovereign nations have asserted control over data about their members.^47^

An additional question is who determines what qualifications are needed to access the data and who verifies that those are satisfied. The primary strategy at present for protecting people whose data are utilized is deciding whether potential users must have certain qualifications, such as working at an institution that certifies and accepts responsibility for the investigator’s actions, being faculty at an educational institution, passing a test or otherwise attesting to knowledge about research ethics, signing a data use agreement or certification, and for protected data, submitting a proposal to the Data Access Committee.^46^ These qualifications vary depending on the sensitivity of the data and can limit participation by citizen scientists who increasingly seek to access data for their own research questions.^48^

A specific issue is whether data access should be affected by the identity of the potential user or the intended data use. The HIPAA Privacy Rule, when data are not de-identified, places restrictions on the parties and specific uses for which data can be disclosed and used without individual authorization.^16^ More broadly, the monetization of data by AI companies, particularly through large language models, has raised questions regarding the ability of data use agreements or licenses to restrict how data are used.^49^ Yet, NIH, as a federal sponsor, expects that data sharing not penalize industry parties. The NIH Data Management and Sharing (DMS) Policy^50^ and the NIH Genomic Data Sharing Policy^36^ do not differentiate between commercial and academic investigators; eligibility hinges on being a “qualified investigator” who agrees to the repository’s Data Use Certification. The NIH Research Tools Policy^51^ urges grantees to make NIH-funded reagents and software available to industry “with the fewest encumbrances possible,” reinforcing non-discrimination. Even when datasets are specified as “not-for-profit only” in participants’ informed consent language, the NIH must approve such limitations.

The location of data users is increasingly important—US Department of Justice regulations on bulk sensitive data issued in January 2025,^52^ and more recently in guidance on April 2025—^53–55^ have clarified that health data of US citizens may not willfully be shared with institutions or individuals from or present in specific countries of concern.^56^ Regardless, all access requires some degree of monitoring, and sustainable funding is often lacking.

Another set of questions is whether the investigator can access some or all the data, and if so, whether the data are de-identified and to what standard. AI-READI, for example, has created both controlled-access and “publicly accessible” datasets, although registration and authentication are required for both. Additional application is required for research that is not focused on diabetes.^2^

### Determine under what conditions data and models can leave the repository

The potential for removing data from a repository raises 2 interrelated questions. The first is who can qualify to download data, issues often assessed by Disclosure Review Boards, which typically are distinct from Data Access Committees. Those who are permitted to download data often are required to sign a data use agreement limiting their ability to share or use the data for other purposes.^57^ There are significant questions, however, whether the limitations specified in such agreements are enforceable, and if so, by whom. Penalizing users for misuse of data by cutting off access to the repository for a certain amount of time is unlikely to sufficiently deter misuse.^58^ Notably, neither HIPAA nor the Common Rule create a private right of action for people harmed by privacy breaches,^34^ although some states are beginning to add such private claims to their statutes.^59^ Furthermore, data repositories often lack the capacity and legal tools to monitor or enforce user compliance with license provisions. This is particularly important in Bridge2AI since the NIH has made clear that the DGPs and presumably their institutions are responsible for the sustainability of the datasets they create.

In some cases, investigators are required to conduct their analyses in a trusted research environment, removing only their results. For example, the Program in Clinical Care AI through the CHoRUS Network requires that all analysis of its data be done in its Azure Cloud tenant. They reason that banning unrestricted export (download) of the CHoRUS data resources is needed to comply with the HIPAA Privacy Rule and state-law restrictions. A similar approach is utilized within other large biomedical data generation projects, most notably the All of Us Research Program.^60^ In other programs, the investigator must go to a specific site to access data. This is true for federal health data enclaves, such as the National COVID Cohort Collaborative (N3C)^61^ and Federal Statistical Research Data Centers (FSRDCs),^62^ the latter which require the researcher to submit a query and receive only the results. This strategy also informs federated learning, where AI models are trained across multiple decentralized datasets without exchanging individual-level records.^63^

Even limiting downloading to models may not protect individual-level data enough to comply with privacy principles or requirements.^64^ Research on membership inference attacks reveals that users may be able to determine if a participant’s record is part of the training dataset,^64^ even when the user is inspecting only synthetic data produced by the model.^65^ Moreover, as the number of parameters in an AI model increases, it becomes more difficult to determine if the training data are embedded in the model and hence extractable. GPT-2 and GPT-4, for example, have been shown to memorize and regurgitate training data based on specific prompts.^66^

## Conclusions

Managing how data are collected, stored, shared, and used is complex and involves all stages of the AI model/product development process. In the United States, the steps in data collection and use involve various actors, each constrained in various ways by law, funding, ethical norms, patterns of practice, and their own interests. Not surprisingly, decision making has usually defaulted to those with the most power, including sponsors and investigators. More effort, however, is needed to identify and address the interests of patients and research participants, individually and collectively, to ensure that the research meets their needs and concerns. Importantly, simply requiring informed consent and IRB oversight in all cases, particularly if these are the only steps taken, will fail to provide the necessary protection, while often coming at substantial cost to the robustness and generalizability of models derived from study data. A crucial first step will be requiring sponsors, institutions, and researchers to engage thoughtfully to the growing body of evidence of the public’s concerns,^67^ including research being conducted by the DGPs.^15^^,^^68^ Future work may include assessing the value of such strategies as deliberative engagement^69^ and including participants on bodies charged with promoting responsible collection and use of data in repositories.^70^^,^^71^

While all actors are responsible for the ethical development of AI, academic medical centers (AMCs), in particular, play a pivotal role in the governance and stewardship of data.^14^^,^^72^ They operate and are accountable in several regulatory and social spheres, from clinical to quality improvement to research. They are public-facing and so have incentives to avoid liability, to promote public trust, and to respond to the peer pressure of other institutions, such as the University of California, that are developing data governance processes.^73^ Relying solely on internal processes will not suffice. Rather, these institutions should focus on providing the infrastructure needed to maintain and monitor these resources and on ensuring that research conducted using data about their patients and research participants meets the needs for health care and protection of all patients. Moreover, individual projects must devote more funding, capacity, and effort to assessing the public’s input at various stages of the process of data collection and use. This will require a commitment by sponsors to provide the time and resources needed to increase capacity among all involved in the creation and governance of health data repositories used for AI.

## Data Availability

There are none to share.
