# Correction to: Biomedical data repositories require governance for artificial intelligence/machine learning applications at every step

**DOI:** 10.1093/jamiaopen/ooaf173

**Published:** 2026-02-23

This is a correction to: Ellen Wright Clayton, Susannah Rose, Camille Nebeker (et al), Biomedical data repositories require governance for artificial intelligence/machine learning applications at every step, *JAMIA Open*, Volume 8, Issue 6, December 2025, https://doi.org/10.1093/jamiaopen/ooaf134

The last name of the third author has been corrected in the article to read: “Camille Nebeker”.

